# An Injury Prevention Programme in Physical Education Teacher Education Students: Process Evaluation Using the RE-AIM Sports Setting Matrix

**DOI:** 10.1155/2024/5717748

**Published:** 2024-09-12

**Authors:** Lennert Goossens, Greet Cardon, Erik Witvrouw, Evert A. L. M. Verhagen, Dirk De Clercq

**Affiliations:** ^1^ Department of Health and Biomedical Sciences Loyola University Andalucía, Sevilla, Spain; ^2^ Department of Movement and Sports Sciences Ghent University, Ghent, Belgium; ^3^ Department of Rehabilitation Sciences Ghent University, Ghent, Belgium; ^4^ EMGO Institute for Health and Care Research Department of Public and Occupational Health VU University Amsterdam Medical Centre, Amsterdam, Netherlands

## Abstract

This study aimed to evaluate the feasibility and effectiveness of an injury prevention programme for Physical Education Teacher Education (PETE) students, consisting of an injury awareness module and implementing prevention strategies during intracurricular lessons. Participants from four PETE programmes formed the intervention group (*n* = 4 programme directors, *n* = 38 sports lecturers, *n* = 859 students), while those from four other programmes were the controls (*n* = 4 programme directors, *n* = 34 sports lecturers, *n* = 721 students). Programme directors and sports lecturers received a three-hour workshop on sports injury prevention. The feasibility and effectiveness of the intervention were evaluated following the RE-AIM Sports Setting Matrix. Reach, adoption, and implementation of the prevention strategies were high, but implementation of the awareness module was moderate, ranging from 25% to 75%. Maintenance in terms of intentions ranged from 25% to 75% for aspects of the awareness module and averaged 68% for the prevention strategies. Significantly more static stretching (*p*=0.029), dynamic stabilisation (*p* < 0.001), and core stability (*p*=0.001) were implemented in the intervention group compared to the control group. Injury prevention behaviour and knowledge in students did not increase after the intervention. In conclusion, moderate feasibility of an injury prevention intervention for PETE students was found. Sports lecturers implemented prevention strategies in their lessons frequently, but future interventions should develop more dissemination initiatives.

## 1. Introduction

Physical education teacher education (PETE) students spend numerous hours on the practice of sports to cover a sustainable range of the many types of sports available [1]. The reported incidence of sports injuries in PETE students ranges from 0.7 to 11.7 injuries/1000 hours of sports participation [2–6]. Most of these injuries occur to the lower limbs and incur during noncontact situations [7]. Injured PETE students miss numerous sports classes and hours of practice [3] and experience physical discomfort and negative consequences on their sports and professional career [8, 9]. In addition, PETE students constitute the near future of physical education (PE) and sports because they will teach PE in schools and/or be engaged in sports training. The detrimental effects of a PE teacher's extensive injury history have been shown before [9], with more PE teachers than referents having to change work or work tasks because of injuries. Injury prevention demands special attention in PETE students because of the potential health consequences and impact on their future professional careers.

Prevention programmes for PETE students should be multifactorial (combining several prevention strategies) and executed around three times per week [10]. The only multifactorial injury prevention programme for PETE students that could be located in the literature is the “No Gain With Pain” (NGWP) programme [11]. This programme was developed based on efficacious sports injury prevention programmes from the literature while considering PETE students' epidemiological and aetiological sports injury data [10]. It consisted of an awareness module and the implementation of prevention strategies in the sports lessons. To increase adherence, NGWP was embedded in the regular PETE programme by the sports lecturers, and after the implementation of NGWP, injury incidence in PETE students was reduced by 20% [11].

To have an actual impact on public health, sports injury prevention programmes should be adhered to during real-world implementation. Determining the feasibility and effectiveness by means of the process evaluation of such a real-world implementation is therefore necessary [12]. A process evaluation can unravel what happened in practice and inform future implementation. Finch and Donaldson [13] proposed the Reach Effectiveness Adoption Implementation Maintenance Sports Setting Matrix (RE-AIM SSM) to evaluate sports injury prevention interventions. The RE-AIM SSM has received considerable attention in the recent literature, but few studies have included all dimensions of the framework [14]. In the PETE context, it has not been employed at all. To achieve effectiveness in terms of injury incidence reduction, intervention adherence is essential [15]. One of the determinants of this prevention-related behaviour is prevention-related knowledge [16].

In this current study, we implemented the NGWP programme [11] in various real-world settings (professional bachelor's degree in PETE programmes). Our first aim was to evaluate aspects of feasibility (reach, adoption, implementation, and maintenance) of NGWP in PETE programmes on various levels (programme directors, sports lecturers, and students). Our second aim was to determine the effectiveness of NGWP on injury prevention-related behaviour and knowledge of PETE sports lecturers and PETE students.

## 2. Materials and Methods

### 2.1. Study Design and Participants

A cluster randomised controlled trial was conducted during one school year. Participants were programme directors and sports lecturers (the target of the researcher-delivered intervention) and 1st- to 3rd-year students (targeted health beneficiaries) from professional bachelor's degree in PETE programmes in Flanders (Belgium). First, all programme directors of PETE programmes in Flanders (*n* = 14) were invited to participate in the study. Eight PETE programmes (57%) confirmed participation and were assigned to the intervention (*n* = 4) or control group (*n* = 4) by a random number generator. PETE programmes of the intervention group were asked to implement NGWP for one school year, while the regular PETE programme was executed in the control group. Sports lecturers employed in PETE programmes of both intervention and control groups were excluded from the study to avoid contamination. All programme directors (*n* = 8), sports lecturers (*n* = 72), and students (*n* = 1,580) of the participating PETE programmes signed an informed consent form. The ethical committee of the Ghent University Hospital approved the protocol (B670201215484).

### 2.2. Intervention

**Figure 1:**
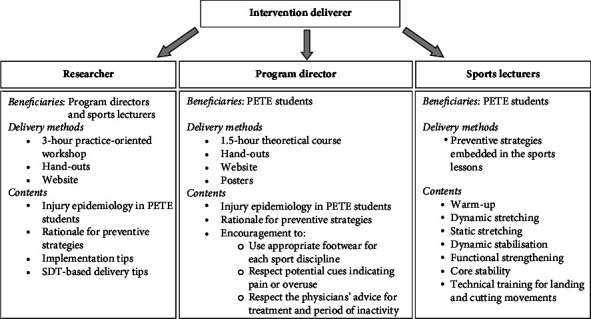
Schematic overview of the intervention. Adapted and reprinted from Goossens et al. [11] by permission of the publisher (Taylor and Francis Ltd). PETE = physical education teacher education; SDT = self-determination theory.

A schematic overview of the intervention can be found in Figure 1. The intervention consisted of a researcher-delivered workshop and an awareness module delivered by the programme directors. Sports lecturers were asked to implement as many prevention strategies as possible during their intracurricular lessons. According to the self-determination theory (SDT) [17], behaviours are regulated by the desire to satisfy the innate psychological needs of autonomy, relatedness, and competence. Therefore, sports lecturers were stimulated to apply the concepts of SDT to enhance students' adherence.

### 2.3. Researcher-Delivered Workshop

Before the start of the school year, programme directors and sports lecturers of the intervention group attended the researcher-delivered three-hour practice-oriented workshop to enhance injury prevention-related knowledge and stimulate preventive behaviour. The workshop included information about injury epidemiology in PETE students and injury prevention strategies (warm-up, dynamic and static stretching, dynamic stabilisation, functional strengthening, core stability, and technical training for landing and cutting movements). Guided by SDT, examples and guidelines were given on implementing these strategies in the sports lessons (e.g., offer exercise variety and differentiation, offer freedom of choice, and determine challenging but attainable goals). Finally, more “passive” prevention strategies were highlighted: appropriate footwear, attention to potential cues indicating pain or overuse, the importance of consulting a sports physician in case of injury, and respect for the physician's recommendations.

### 2.4. Awareness Module

Programme directors were asked to deliver the awareness module: (1) to organise a theoretical injury prevention course for the students and (2) to distribute the supporting hand-outs. The course consisted of information about the most frequently occurring injuries in PETE students and the rationale for each prevention strategy. Moreover, the abovementioned “passive” prevention strategies were highlighted. Programme directors were also asked (3) to hang posters about sports injury prevention on campus and (4) to inform the students about a supporting intervention website. The researcher delivered all supporting materials—digital presentation, hand-out file, printed posters, and website address—to the programme directors. The hand-out file and posters can be found in Appendix 1.

### 2.5. Measurement Instruments

Aspects of feasibility and effectiveness of NGWP were evaluated following the RE-AIM SSM. Several new custom-made questionnaires were developed based on the RE-AIM Model Dimension Items Checklist [18], to ensure that all five dimensions of the RE-AIM framework were covered (Table 1). The *Characteristics Questionnaires* and *Implementation and Maintenance Questionnaires* included both open- and closed-ended questions. The *Behaviour and Knowledge Questionnaires* (BKQ) included questions about applying prevention strategies on a 5-point Likert scale ranging from “never” to “always,” 4 true/false questions and 11 multiple choice questions about injury prevention-related knowledge. The questionnaires' understanding and readability were improved following feedback from two experienced delivery agents (one programme director and one sports lecturer) not involved in the study.

All programme directors completed a Programme Characteristics Questionnaire before the intervention, and those of the intervention group completed an Implementation and Maintenance Questionnaire after the intervention.

All sports lecturers completed a Sports Lecturer Characteristics Questionnaire and a Behaviour and Knowledge Questionnaire (BKQ_SL) before and after the intervention. Furthermore, they registered programme adherence online weekly. Based on their registrations, the average number of injury prevention sessions the students received per week was calculated. Additionally, sports lecturers of the intervention group completed an Implementation and Maintenance Questionnaire after the intervention.

Students of the intervention and control group completed a Behaviour and Knowledge Questionnaire (BKQ_St) before and after the intervention. Moreover, the intervention group students completed an Implementation and Maintenance Questionnaire after the intervention.

To test the reliability of the BKQ, a separate sample of 18 4th-year PETE students completed the questionnaire twice with a time interval of 1 week. The reliability of the “self-reported behaviour” questions was assessed by calculating the intraclass correlation coefficients (ICC). All items scored at least “average to good” (>0.40) on the Fleiss reliability scale [19], and the average score was “excellent” (average single measures ICC = 0.75 ± 0.14; range: 0.44). The reliability of the “knowledge” questions was assessed by calculating percentage agreement on both occasions. Percentage agreement above 70% led to inclusion in the final analyses [20]. Of the 15 questions, all except three had a percentage agreement above 70% (average 83.6 ± 10.8% agreement; range: 29.50). Those three were excluded from the final analysis, so the highest possible score for “knowledge” was 12.

### 2.6. Data Collection

All participants received a link by e-mail, directing them to the online questionnaires. Reminders were sent by e-mail to nonresponders after 7 and 14 days. Moreover, sports lecturers received a weekly reminder e-mail with a link to register online, which prevention strategies they had implemented in their lessons during the past week.

### 2.7. Statistical Analyses

Comparability between the groups regarding the number of sports lecturers, students, and weekly sports lessons was tested using independent samples *t*-tests. Mixed Models ANOVA (Wilks' Lambda) was used to test differences between the intervention and control groups in pre- and post-self-reported behaviour and knowledge changes. A Pearson chi-square test was used to test differences in prevention strategy implementation between the intervention and control groups' sports lecturers. The significance level was set at *α*<0.05, and IBM SPSS statistics 29 was used.

## 3. Results

### 3.1. Reach

Eight out of 14 PETE programmes in Flanders (57%) participated in the study. Of the six nonparticipating PETE programmes, three did not participate due to a lack of time, two did not react after several requests, and one did not want to interfere with another injury prevention programme administered at the time. None of the eight participating PETE programmes dropped out during the study. There were no significant differences regarding the number of sports lecturers, students, and weekly sports lessons between the participating and nonparticipating PETE programmes, nor between the intervention and control group. Before the start of the study, three nonparticipating (50%) and two participating (25%) PETE programmes of the control group already had a structured injury prevention policy, including warm-up, stretching, strength, stabilisation, and technical training. For two nonparticipating (33%) and two participating (25%) PETE programmes (one intervention group; one control group), “injury prevention” was already included in the mission of the programme before the start of the study.

The study reached 72 out of 124 sports lecturers (58%) and 1,580 out of 2,665 students (59%). One sports lecturer was excluded from the study because he was employed by PETE programmes of both the intervention and control groups. Sports lecturers of the intervention and control groups were not significantly different regarding age and sports teaching experience (Table 2).

### 3.2. Effectiveness

After the intervention, 75% of the programme directors and the sports lecturers believed that NGWP could reduce the incidence of sports injuries. One programme director (25%) and 47.8% of the sports lecturers believed that NGWP has the potential to improve academic results.

Weekly registrations showed that sports lecturers in the intervention group implemented significantly more static stretching, dynamic stabilisation, and core stability in the sports lessons than those in the control group. Interaction effects showed no significantly different pre-post changes between the intervention and control groups. However, trends towards a higher increase in the intervention group were observed for dynamic stabilisation, functional strengthening, and knowledge.

Interaction effects revealed a significantly greater increase of knowledge in students of the intervention group compared to the control group but no significant differences in self-reported behaviour. However, trends towards a higher increase in the intervention group were observed for core stability (*p*=0.078), using appropriate footwear (*p*=0.069), and respecting potential cues indicating pain or overuse (*p*=0.089) (Table 3).

### 3.3. Adoption

All four PETE programmes in the intervention group (100%) adopted the intervention. Of 38 sports lecturers, 33 (87%) attended the workshop. The five nonattending sports lecturers had other professional duties. Attending and nonattending sports lecturers were not significantly different regarding age (*p*=0.547) and sports teaching experience (*p*=0.645).

### 3.4. Implementation

An overview of the implementation of NGWP is provided in Table 4. Of four programme directors, one (25%) organised the theoretical course in a single session, two (50%) did it in various sessions, and one (25%) did not organise the theoretical course because the contents were already covered in various PETE subjects. None of them (0%) indicated that the intervention costed extra time or money.

Thirty-six percent of the sports lecturers indicated that the intervention required an extra investment of time, with, on average, 15 minutes weekly. Popular implementation strategies included “exercise execution during instruction moments,” “exercise execution while students wait for their turn,” “ask help of internship students,” “provide additional exercises for injured students,” and “organise additional lessons.” Moreover, several lecturers adapted the implementation in their lessons to the implementation of fellow lecturers on the same day or in the same week. Practical barriers to implementation included “time restrictions” and “practical difficulties in some sports (e.g., swimming).” Programme content-related barriers included “the workshop was limited in time and profoundness,” “the information and supporting didactical material for implementation was limited,” “the information on the website was limited,” and “problems remembering workshop information.”

After the intervention, 17% of the students remembered the posters, 4% remembered the theoretical course, 6% remembered the hand-outs, and 6% remembered visiting the website.

### 3.5. Maintenance

One programme director (25%) intended to deliver the complete awareness module in the subsequent school year. Three (75%) had the intention to inform about the website, two (50%) to hang the posters, two (50%) to organise the theoretical course, and one (25%) to distribute the hand-outs in the subsequent school year. However, the two programme directors, without intention to organise the theoretical session, indicated that the contents would be included in other PETE subjects. Three programme directors (75%) intended to include “injury prevention” in their programme's mission in the subsequent school year.

Eighty-three percent of the sports lecturers intended to implement warm-up, 54% dynamic stretching, 46% static stretching, 71% dynamic stabilisation, 63% functional strengthening, 83% core stability, and 75% technical training for landing and cutting movements in the subsequent school year. To facilitate future implementation, several lecturers suggested encouraging independent execution of static stretching after finishing class.

Of all students, 69% would maintain warm-up within the programme, 55% dynamic stretching, 34% static stretching, 46% dynamic stabilisation, 51% functional strengthening, 40% core stability, and 40% technical training for landing and cutting movements in the subsequent school year.

## 4. Discussion

The study results showed moderate feasibility of the NGWP injury prevention programme. Reach, adoption, and implementation of the prevention strategies were high, with significantly more implementation of static stretching, dynamic stabilisation, and core stability in the intervention group compared to the control group. Implementation of the awareness module by programme directors ranged from 25% to 75%. Maintenance in terms of intentions ranged from 25% to 75% for aspects of the awareness module and averaged 68% for the prevention strategies. Injury prevention behaviour and knowledge in students did not increase after the intervention.

### 4.1. Reach

Forty-three percent of the contacted PETE programmes refused to participate in this study. Other studies implementing a sports injury prevention programme in a multisport educational setting reported nonparticipation rates ranging from 7 to 92% [21–24], but none of these reported motivations for declined participation. In the current study, one PETE programme declined to implement the programme since another injury prevention programme was already embedded in the curriculum. Three PETE programmes refused because they had experienced a high time investment for participation in scientific studies before. Future studies should emphasize that the researcher-delivered workshop is concise and that the required time commitment to injury prevention during the intracurricular lessons is limited. Two PETE programmes repeatedly ignored the invitation to participate. Time constraints or lack of perceived utility of injury prevention are possible reasons. Hence, the necessity and efficacy of injury prevention for PETE students should be highlighted when presenting NGWP.

### 4.2. Effectiveness

Efficacy of No Gain With Pain has been shown before, with an injury incidence reduction of 20% [11]. Real-world effectiveness in terms of injury incidence could thus be expected. Yet, in the current study, injuries were not registered. As prevention adherence is essential for its effectiveness [15], the programme directors' and sports lecturers' belief of NGWP's potential for success was evaluated. Moreover, sports lecturers and PETE students evaluated injury prevention-related behaviour and knowledge.

Seventy-five percent of programme directors and sports lecturers believed NGWP has the potential to reduce the incidence of sports injuries. This is comparable to the study of Dix et al. [25], where 74% of football coaches believed in the efficacy of a knee injury prevention programme. However, higher percentages were reported by Cornelissen et al. [26], with 92% of board members and 85% of coaches believing in the efficacy of a hockey injury prevention programme, and Stensø at al. [27] where all delivery agents believed in the efficacy of an adductor prevention programme for football players.

In sports lecturers, analysis of the weekly registrations showed that static stretching, dynamic stabilisation, and core stability were implemented significantly more in the intervention group than in the control group. However, despite increased values for all active prevention strategies except for warm-up in the intervention group compared to similar or lower values in the control group, no significantly different pre-post changes in self-reported behaviour between the intervention and control groups were observed. Several reasons could explain this lack of significant increases. For time constraints, implementation in all sports lessons (= score 5) or more than half of the sports lessons (= score 4) seemed impossible for all active prevention strategies except for warm-up. Nonetheless, sports lecturers of the intervention group adapted the implementation in their lessons to the implementation of fellow lecturers to execute each active strategy at least once every week. For this reason, implementation scores remained between “in less than half (= score 2) and in half (= score 3) of the sports lessons,” with a possible implementation range of 1%–50% of the sports lessons. In addition, we observed a (nonsignificantly) increased score in the intervention group compared to a lower score in the control group for knowledge after the intervention. Consequently, more knowledge about the “perfect” implementation of prevention strategies in the intervention group might have led to a lower perception of the implementation and a diminished effect size.

In students, the significantly increased knowledge in the intervention group compared to the control group was mainly caused by a decreased score in the control group, hypothesizing only a small effect of the NGWP intervention. Future studies should schedule an extra measurement immediately after the theoretical injury prevention course to investigate whether this is due to a lack of retention of the information 10 months after the course. Since only 6% of the students visited the website, other reminders and knowledge transfer forms should be investigated. Furthermore, despite the behavioural approach of the NGWP programme, self-reported behaviour remained similar in both the intervention and control groups after the intervention. The implementation of injury prevention strategies during the intracurricular sports lessons possibly resulted in a perception of decreased necessity to execute them during extracurricular sports activities. Also, the program directors' rather low implementation rate of several aspects of the awareness module might have caused low perceived utility of injury prevention. To better understand students' injury preventive behaviour, future studies should investigate the effect of NGWP on behavioural determinants like autonomous motivation, attitude towards the behaviour, subjective norms, perceived behavioural control, and behavioural intentions. On the other hand, prevalues of self-reported behaviour in both groups showed that PETE students execute active prevention strategies in half of the sports activities, albeit in the absence of a structured injury prevention programme. This is consistent with Bliekendaal et al. [7], who found that PETE students frequently apply prevention strategies without a structured injury prevention programme.

### 4.3. Adoption

Maximum adoption by programme directors was achieved. Adoption by sports lecturers was high (87%) compared to other studies, including a researcher-delivered intervention [26, 28]. Only a few sports lecturers did not attend the workshop, and this was for reasons beyond their control. Probably, no important differences existed between attending and nonattending sports lecturers. The organisation of a specialisation course for teaching staff within the premises of the institution thus seems a good approach.

### 4.4. Implementation

The programme directors' implementation of the awareness module ranged from low to high. Only one programme director did not organise the theoretical course because the contents were already covered in other PETE subjects. Distribution of the posters (2/4) and hand-outs (1/4) was low, possibly because of costs and time related to printing the hand-outs and hanging the posters. In comparison, Lindblom et al. [28] reported that 41% of clubs used the material provided by the researchers. Future research should emphasize the importance of raising PETE students' awareness of injury prevention.

Goossens et al. [10] reviewed the literature for injury prevention programmes and concluded that sports injury prevention in PETE students should be executed around three times per week to be efficacious. In the current study, the execution of warm-up, technical training for landing and cutting movements, and dynamic stretching reached this recommendation with six, four, and three sessions per week, respectively. The other active prevention strategies were executed two times per week. These implementation rates are relatively high compared to other studies implementing a sports injury prevention programme in a multisport educational setting. In the study of Emery et al. [23], 78% of the students participated in at least two weekly sessions and programme efficacy was found in females only. Likewise, Slauterbeck et al. [24] reported 32% of the students performing the injury prevention programme at least twice a week, but no programme efficacy was found. These results underscore the importance of programme execution around three times per week. Furthermore, sports lecturers in the present study reported an extra time investment of less than 15 minutes weekly. These findings confirm that, in an educational setting with numerous weekly sports sessions, injury prevention should not necessarily be implemented through a standardized warm-up as it is mostly applied in team sports such as soccer [29]. The approach of NGWP, with examples and guidelines on implementing prevention exercises into the regular PETE programme, seems feasible. However, suggestions from various sports lecturers to expand both the workshop and didactical materials should be considered.

Compared to other interventions using delivery agents, few students in the current study remembered the awareness module (4–17%). Sewry et al. [30] found that 34% of South African rugby players had never heard of the BokSmart Safe Six programme. In the study of Harøy et al. [31], 30% of football players reported knowing the content of an adductor strengthening programme. Furthermore, in a survey of Nigerian football players, 21% were aware of the FIFA 11+ programme [32]. This is remarkable as PETE students in the study by Bliekendaal et al. [7] found knowledge essential to implement prevention strategies within their daily routines. Dissemination materials such as posters, hand-outs, and websites are possibly outdated, and future interventions should focus on dissemination initiatives through mobile applications and social media.

### 4.5. Maintenance

All PETE programme directors intended to teach injury prevention-related content in the subsequent school year. This is higher than Lindblom et al. [28] who found that only 57% of football club personnel intended to educate their coaches in the upcoming season. Sports lecturers' intention to implement the different active prevention strategies averaged 68%. Comparable results were found by Cornelissen et al. [26] and Andersson et al. [33], where 76% of hockey coaches and 71% of handball coaches, respectively, agreed to use (parts of) an injury prevention programme in the upcoming season. On the other hand, all football coaches in the study of Lindblom et al. [28] agreed to prioritize the injury prevention programme in the upcoming season. Students' intention to maintain the different active prevention strategies in the programme averaged 48%. Likewise, handball players in the study of Andersson et al. [33] showed lower intentions (57%) than their coaches to implement the injury prevention programme in the upcoming season. On the other hand, 65% of football players in the study of Harøy et al. [31] intended to perform an injury prevention programme in the upcoming season. Future studies should investigate whether the relatively low student intention in the current study is related to the programme's relevance, time investment, entertainment level, or other factors.

### 4.6. Strengths and Limitations

This study was the first to perform a process evaluation of the real-world implementation of an injury prevention programme for PETE students. The NGWP programme applied a multilevel intervention approach, and the RE-AIM SSM guided the process evaluation. However, future studies should prospectively register injuries. Moreover, the large drop-out at the student level might have biased the postsurvey results. In addition, a follow-up questionnaire in the subsequent year would measure programme maintenance more accurately.

## 5. Conclusion

The feasibility of the NGWP injury prevention programme was moderate. More than half of the PETE programmes accepted to participate in the study, and their adoption of NGWP was high. Implementation of the prevention strategies by the sports lecturers was high, as well as the programme directors' delivery of the theoretical course. However, the programme directors' dissemination of other aspects of the awareness module was rather low. Sports lecturers in the intervention group implemented significantly more prevention strategies than those in the control group, but no such differences were observed in students. Injury prevention knowledge did not increase significantly after the intervention. Intentions to maintain programme implementation in the subsequent year were high among sports lecturers and moderate among students. This study provides suggestions for NGWP adjustments to increase programme feasibility. Future programme versions should include other dissemination initiatives to increase students' awareness and should expand both the researcher-delivered workshop and didactical materials. Future studies should prospectively register injuries and investigate sports injury-related behavioural determinants.

## Figures and Tables

**Table 1:** Overview of the dimensions and levels of the RE-AIM SSM and corresponding outcome measures and measurement instruments.

Dimension	Level	Outcome measure	Measurement instrument
Reach	Organization	The proportion of PETE programmes that participated in the study	—
		Characteristics of participating vs. nonparticipating PETE programmes: # sports lecturers, # students, average # weekly sports lessons, presence of a structured injury prevention policy, presence of injury prevention in the mission of the PETE programme	Programme characteristics' questionnaire
	Staff	The proportion of sports lecturers who participated in the study	—
		Characteristics of sports lecturers in intervention vs. control groups: age, sports teaching experience	Sports lecturer characteristics questionnaire
	Student	The proportion of students who participated in the study	—

Effectiveness	Organization	Belief in NGWP for injury prevention and study results (yes or no)	Implementation and maintenance questionnaire for programme directors
	Staff	Belief in NGWP for injury prevention and study results (yes or no)	Implementation and maintenance questionnaire for sports lecturers
		Differences between intervention and control groups in pre-post changes of self-reported behaviour of prevention strategies and injury prevention-related knowledge	Behaviour and knowledge questionnaire for sports lecturers
		Differences in programme adherence between intervention and control groups	Weekly online registration after invitation by e-mail
	Student	Differences between intervention and control groups in pre-post changes of self-reported behaviour of prevention strategies and injury prevention-related knowledge	Behaviour and knowledge questionnaire for students

Adoption	Organization	The proportion of PETE programs in the intervention group that adopted the intervention	—
	Staff	The proportion of sports lecturers in the intervention group who attended the workshop	—
		Characteristics of sports lecturers who adopted vs. those who did not adopt the intervention: age, sports teaching experience	Sports lecturer characteristics questionnaire

Implementation	Organization	Delivery, adaptations and costs of the theoretical course, hand-outs, posters, and website	Implementation and maintenance questionnaire for programme directors
	Staff	Adaptations and costs of the implementation of the prevention strategies	Implementation and maintenance questionnaire for sports lecturers
		Percent of lessons in which the sports lecturers implemented each prevention strategy	Weekly online registration after invitation by e-mail
	Student	If students remembered the theoretical course, hand-outs and posters and if they visited the website	Implementation and maintenance questionnaire for students

Maintenance	Organization	Maintenance of the theoretical course, hand-outs, posters, and website in the subsequent school year	Implementation and maintenance questionnaire for programme directors
		Intention to include injury prevention in the mission of the PETE programme in the subsequent school year	
	Staff	Maintenance of the prevention strategies in the subsequent school year	Implementation and maintenance questionnaire for sports lecturers
	Student	Maintenance of the prevention strategies in the subsequent school year	Implementation and maintenance questionnaire for students

PETE = physical education teacher education; NGWP = no gain with pain.

**Table 2:** Characteristics of the intervention, control, and nonparticipating groups.

	IG	CG	*t* ^*a*^	*p*	Nonparticipating	*T* ^*b*^	*p*
# of PETE programmes	4	4			6		
# of sports lecturers	38	34	0.705	0.521	52	−0.266	0.795
# of sports lecturers completing the BKQ_SL before (and after) the study	33 (26)	9 (7)			—		
Average age (±SD) of the sports lecturers after drop-out	40 ± 9	39 ± 10	0.33	0.744	—		
Average years of experience as a sports teacher (±SD) after drop-out	15 ± 9	15 ± 10	−0.27	0.979	—		
# of students	859	721	0.452	0.667	1085	−0.275	0.788
# of students completing the BKQ_St before (and after) the study	371 (109)	145 (84)			—		
Average weekly hours of sports lessons	8	7	0.889	0.408	8	0.575	0.576

IG = intervention group; CG = control group; BKQ_SL = behaviour and knowledge questionnaire for sports lecturers; BKQ_St = behaviour and knowledge questionnaire for students; PETE = physical education teacher education; SD = standard deviation; ^a^intervention vs. control groups; ^b^participating vs. nonparticipating groups.

**Table 3:** Prevention strategy implementation (weekly registrations), self-reported behaviour on a 5-point Likert scale, and knowledge sum score (maximum score = 12).

	Sports lecturers											Students						
	Prevention strategy implementation				BKQ sports lecturers							BKQ students						
	IG (*n* = 33)	CG (*n* = 9)			IG (*n* = 26)		CG (*n* = 7)					IG (*n* = 109)		CG (*n* = 84)				
	% of sports lessons		Chi^2^	*p*	Pre	Post	Pre	Post	*F*	*p*	*η*_*p*_^2^	Pre	Post	Pre	Post	*F*	*p*	*η*_*p*_^2^
*Self-reported behaviour*																		
Warm-up	87.4	87.7	0.008	0.928	4.70	4.55	4.88	4.88	0.091	0.765	0.003	3.92	3.87	4.17	4.15	0.022	0.882	<0.001
Dynamic stretching	43.5	40.6	0.243	0.622	2.15	2.75	2.50	2.88	0.176	0.678	0.007	3.14	3.13	3.21	3.30	0.261	0.610	0.002
Static stretching	22.4	12.3	4.748	0.029^a^	1.55	1.95	2.13	2.00	2.081	0.161	0.074	2.76	2.80	3.23	3.15	0.272	0.603	<0.001
Dynamic stabilisation	34.1	13.2	15.65	<0.001^b^	1.85	2.55	2.38	2.25	3.478	0.074	0.118	2.56	2.73	2.57	2.72	0.013	0.910	0.001
Functional strengthening	22.4	17.0	1.286	0.257	2.20	2.85	2.63	2.50	4.165	0.052	0.138	2.91	2.98	3.19	3.17	0.157	0.693	0.023
Core stability	36.0	17.9	11.006	0.001^b^	2.05	2.65	2.25	2.38	1.704	0.203	0.062	2.97	2.85	3.02	3.26	3.147	0.078	0.001
Technical training	52.3	60.4	1.852	0.174	2.95	3.35	4.00	4.13	0.171	0.683	0.007	3.70	3.51	3.98	3.74	0.079	0.779	0.002
Appropriate footwear	—	—	—	—	4.80	4.50	4.75	4.50	0.032	0.860	0.001	3.95	4.06	4.30	3.96	3.362	0.069	0.025
Respect cues	—	—	—	—	4.50	4.55	4.63	4.50	0.288	0.596	0.011	3.47	3.42	3.87	3.47	2.943	0.089	0.022
Consult sports physician	—	—	—	—	4.60	4.80	4.75	4.75	0.646	0.429	0.024	3.57	3.60	3.74	3.66	0.301	0.584	0.002
Respect treatment	—	—	—	—	—	—	—	—	—	—		3.98	3.83	4.23	3.72	2.669	0.105	0.020
Respect inactivity	—	—	—	—	—	—	—	—	—	—		3.83	3.69	3.79	3.51	0.386	0.535	0.003
Knowledge	—	—	—	—	6.54	8.19	7.43	7.14	3.397	0.075	0.099	6.18	6.70	7.48	5.95	17.870	<0.001^b^	0.101

CG = control group; IG = intervention group; BKQ = behaviour and knowledge questionnaire; ^a^ = significantly different on *α* = 0.05-level; ^b^ = significantly different on *α* = 0.01-level.

**Table 4:** Implementation of no gain with pain.

Level	Strategy	Implementation
Setting level (*n* = 4)	Posters delivered	2 programme directors (50%)
	Website delivered	3 programme directors (75%)
	Theoretical course delivered	3 programme directors (75%)
	Hand-outs delivered	1 programme director (25%)

Staff level (*n* = 33)	Warm-up	6 sessions/week^a^
	Dynamic stretching	3 sessions/week^a^
	Static stretching	2 sessions/week^a^
	Dynamic stabilisation	2 sessions/week^a^
	Functional strengthening	2 sessions/week^a^
	Core stability	2 sessions/week^a^
	Technical training	4 sessions/week^a^

Student level^b^	Remembered the posters (*n* = 541)	17.1% of students
	Visited the website (*n* = 801)	6.3% of students
	Remembered the theoretical course (*n* = 699)	4.3% of students
	Remembered the hand-outs (*n* = 160)	5.7% of students

^a^Average number of sessions weekly. ^b^*n* on the student level depended on the number of programme directors delivering the strategy.

## Data Availability

The data that support the findings of this study are available from the corresponding author upon reasonable request.

## Supplementary Materials

Supplementary Materials NGWP posters and hand-outs.
