# Supplementary material for: An Injury Prevention Programme in Physical Education Teacher Education Students: Process Evaluation Using the RE-AIM Sports Setting Matrix
Source: Transl Sports Med. 2024 Sep 12;2024:5717748. doi: 10.1155/2024/5717748 (PMC11412748; doi:10.1155/2024/5717748)
Supplement: Supplementary Materials — NGWP posters and hand-outs. [file 5717748.f1.pdf]

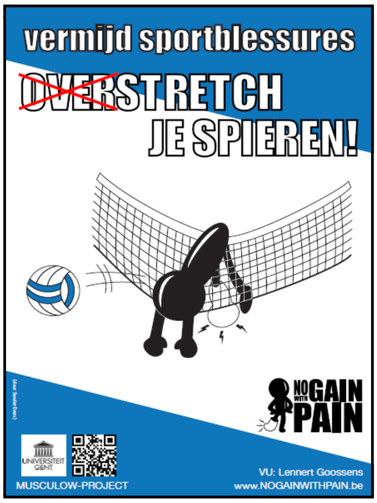
vermijd sportblessures
~~OVER~~STRETCH
JE SPIEREN!
NO GAIN WITH PAIN
UNIVERSITEIT GENT
MUSCULOW-PROJECT
VU: Lennert Goossens
www.NOGAINWITHPAIN.be

1

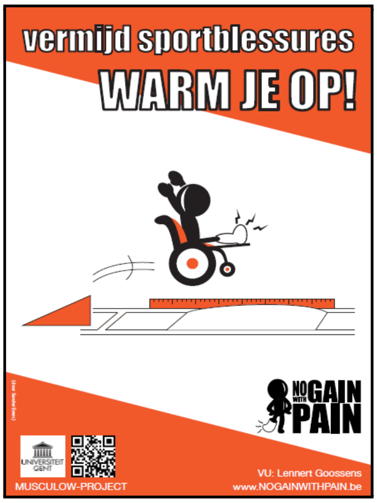
vermijd sportblessures
WARM JE OP!
NO GAIN WITH PAIN
UNIVERSITEIT GENT
MUSCULOW-PROJECT
VU: Lennert Goossens
www.NOGAINWITHPAIN.be

2

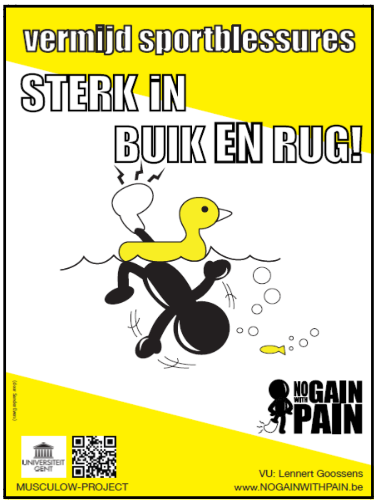
vermijd sportblessures
STERK IN
BUIK EN RUG!
NO GAIN WITH PAIN
UNIVERSITEIT GENT
MUSCULOW-PROJECT
VU: Lennert Goossens
www.NOGAINWITHPAIN.be

3

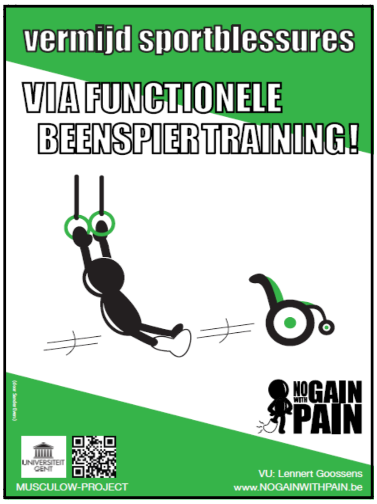
vermijd sportblessures
VIA FUNCTIONELE
BEENSPIERTRAINING!
NO GAIN WITH PAIN
UNIVERSITEIT GENT
MUSCULOW-PROJECT
VU: Lennert Goossens
www.NOGAINWITHPAIN.be

4

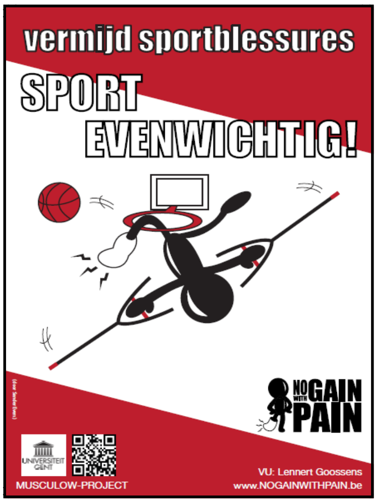
vermijd sportblessures
SPORT
EVENWICHTIG!
NO GAIN WITH PAIN
UNIVERSITEIT GENT
MUSCULOW-PROJECT
VU: Lennert Goossens
www.NOGAINWITHPAIN.be

5

NO GAIN WITH PAIN!

Preventiestrategieën voor blessures aan de onderste ledematen

1

MUSCULOW

1. Epidemiologisch onderzoek blessures bij studenten en leerkrachten LO
   – Online registratie (incidentie)
   – Sportmedische begeleiding
2. Interventie ter preventie van blessures aan de OL bij studenten en leerkrachten LO
   – Implementatie van een actief programma
   – Opvolging blessure-incidentie

2

Blessures aan de OL in cijfers

1 BA academiejaar 2010-2011

- 109 blessures verdeeld over 72 studenten van 128
- Blessurerisico: 0.85 blessures/student/jaar
- 65.1% acuut, 75.2% non-contact, 69,7% nieuw
- 74.3% onderste ledematen

Verdeling blessures bij studenten LO (in %)					
Onderbeen	Knie	Enkel	Schouder	Voet	Bovenbeen
22.9	15.6	15.6	11.9	8.2	6.4

- 34.9% tijdens de lessen op het hilo

3

Blessures aan de OL in cijfers

1 BA academiejaar 2011-2012

- 97 blessures verdeeld over 60 studenten van 102
- Blessurerisico: 0.95 blessures/student/jaar
- 73.2% acuut, 80.4% non-contact, 75.3% nieuw
- 76.3% onderste ledematen

Verdeling blessures bij studenten LO (in %)				
Onderbeen	Bovenbeen	Enkel	Vingers	Knie
23.7	21.6	14.4	12.4	10.3

- 35.1% tijdens de lessen op het hilo

4

Blessures aan de OL in cijfers

1 BA academiejaar 2011-2012

Aandeel sporten opgelopen blessures tijdens de lessen op het hilo

		Frequency	Percent
Valid	atletiek	12	35,3
	basket	1	2,9
	voetbal	4	11,8
	dans	1	2,9
	gymnastiek	8	23,5
	handbal	6	17,6
	volley	2	5,9
	Total	34	100,0

5

NO GAIN WITH PAIN!

➔ Objectief: verminderen van de blessure-incidentie bij bachelor studenten Lichamelijke Opvoeding

➔ Doelgroep: studenten **1ba en 2ba** aan het HILO in AJ 2012-2013
   ➢ Start: Introductiedagen!!

➔ Methode: multiple interventie mbv SDT

➔ Mediatoren:
   ➢ praktijkassistenten HILO
   ➢ Studenten ZELF!!!

6

NO GAIN WITH PAIN!
Design multiple interventie

- Theorieles
- Intrinsieke preventiestrategieën verwerkt in de (opwarmingen van) praktijkvakken
- Aangepast schoeisel
- Vermijden hervalblessures
- Luister naar je lichaam!
- Ook tijdens extracurriculaire sportbeoefening
- Website www.nogainwithpain.be
- Affiches

7

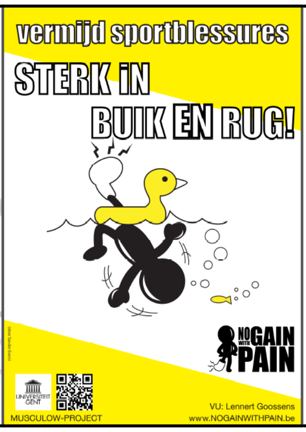

8

NO GAIN WITH PAIN!
Praktisch

- Wekelijkse registratie sportparticipatie en opgelopen blessures
  ➔Link naar registratie via wekelijkse reminder-email
- Retrospectieve bevraging op einde van het academiejaar

9

Strategieën ter preventie van blessures aan de OL

1. Bewustzijnprogramma's: educatieve training
2. Opwarming en cooling-down
3. Stretching
4. Krachttraining
5. Balanstraining
6. Core stability training
7. Multiple interventie
8. Schoeisel aangepast ad sportactiviteit
9. Vermijden van hervalblessures
10. Luister naar je lichaam!

10

1. Bewustzijnsprogramma's: educatieve training

1. Voorste kruisband-blessures (vaak geassocieerd met meniscusscheur)
   - Landen
     - Te weinig knieflexie
     - Te weinig heupflexie
     - Hoge knie valgus (X)
   - Insnijden
     - Hoge dynamische valgus (X-benen)
     - Grote heup adductie
   - Stoppen
     - Hoge knie valgus (X-benen)

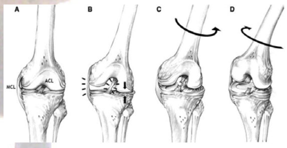

11

Hoge kniebelasting bij initiële contactfase en bij maximale valgus (X-benen) tijdens de landingsfase van een "Drop vertical jump"

Weinig heupflexie

Hoge knie valgus (X-benen)

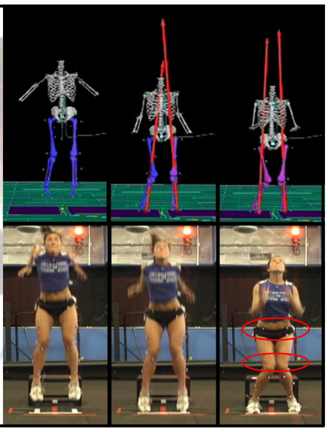

Uit: Myer et al., 2010

12

Criteria	Visual (athletes view)	Yes Left	Yes Right
**Take-off (front view)**			
1. Position of the knee during take-off	Knee movement inside (x-legs)<br>OR<br>Knee movement outside (o-legs)		
2. Position of the ankle during take-off	Ankle tilting inside or outside<br>OR<br>Feet not in the extended of the lower leg		
**Landing (front view)**			
3. Position of knee during landing	Knee movement inside (x-legs)<br>OR<br>Knee movement outside (o-legs)		
4. Controlled landing	Lateral side movements of the knee<br>OR<br>Sudden stops/ stiffening of lower extremity		
5. Position of the ankle during landing	Ankle tilting inside or outside<br>OR<br>Feet not in the extended of the lower leg		
6. Balance	2-feet not simultaneous planted down<br>OR<br>Taking extra step or waving with the arms		
7. Position of shoulder during landing	Feet not planted on shoulder width<br>THUS<br>Feet base smaller or wider than shoulders		
**Landing (side view)**			
8. Trunk during landing	Shoulders before or after knees<br>OR<br>Not maintaining physiological curves of trunk		
9. Position of knee during landing	Knees before toes		
10. Position of the ankle during landing	No rolling off of the feet (toes to heel)<br>OR<br>Landing with flat feet		
11. Maximal ankle angle during landing	To extended position<br>OR<br>X > 90°		
12. Maximal knee angle during landing	To extended position<br>OR<br>X > 130°		

13

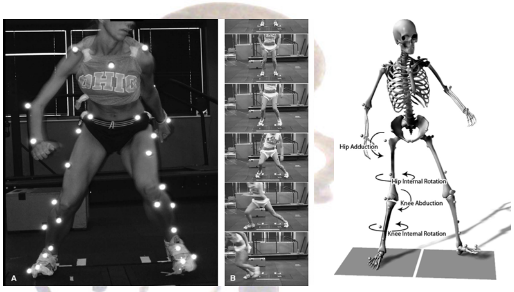

Uit: Imwalle et al., 2009

14

2. Patellofemorale pijn
   - Landen
     - Lage knieflexie
     - Hoge knie valgus (X)
     - Hoge interne rotatie van de heup

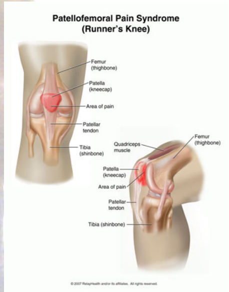

15

3. Enkel verstuiking
   - Insnijden of bijtrekpas
     - Verhoogde plantair flexie (tenen wijzen naar beneden) bij initieel voetcontact

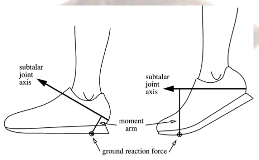

Fig. 2. A view of the foot and ankle in the sagittal plane at touchdown when performing a cutting or side-shuffle movement. The moment arm of the horizontal component of the ground reaction force about the subtalar joint when first contact is made with the heel (left) is much smaller than the moment arm when the foot is plantarflexed and first contact is made at the toe (right).

Uit: Wright et al., 2000

16

Mogelijke oplossingen

- Gebruik van videobeelden
  - Beelden van ongevallen
  - Begrip van blessuremechanismen
- Aanleren van correcte technieken
  - Landingstechnieken
  - Insnijtechnieken
  - …
  - → Hoe risicoposities vermijden?

17

Voorbeelden in de literatuur

Effectiviteit?	Auteur (jaar)	Studie design	Studiegroep
Educatieve training/video			
Effectief	Ettlinger et al. (1995)	Non-RCT	Skiërs
	Jorgensen et al. (1998)	RCT	Skiërs
	Scase et al. (2006)	Non-RCT	Australian Footballers
Niet effectief	Arnason et al. (2005)	RCT	Voetballers

18

2. Opwarming…

- Een opgewarmde spier vereist een hogere mate van rek vooraleer een scheur optreedt (aangetoond bij ratten)
- Na opwarming is er verhoogd bewustzijn van knie gewrichtspositie. Compensatoir mechanisme tgv:
  - Verhoogde gevoeligheid mechanoreceptoren rond knie
  - Meer centraal mechanisme
- Opwarming zorgt voor minder stijfheid na excentrische spieractiviteit (contractie tijdens spierverlenging)

…en cooling-down

- Verlaagt de hartslag en lichaamstemperatuurgradueel
- Afvoer van melkzuur

19

Richtlijnen

- Opwarming mag licht zweten opwekken, zonder vermoeidheid te implementeren
  - Te hoge intensiteit: uitputting van hoge energie fosfaten
  - DUS: inter-individuele verschillen!
- Specifieke opwarming is meest effectief
- 10’ opwarming heeft meer effect op proprioceptie dan 5’ opwarming, 15’ > 10’
- Cooling down: 15’ > 10’ > 5’ voor ↘ kans op blessures

20

Voorbeelden in de literatuur

Effectiviteit?	Auteur (jaar)	Studie design	Studiegroep
Opwarming en cooling-down			
Effectief	Malliou et al. 2007	Non-RCT	Aerobic instructeurs

21

3. Stretching

- Gevolgen van stijve spieren

•Tijdens excentrische activiteit neemt actieve kracht af terwijl passieve krachtwerking toeneemt

•Spierschade kan opgelopen worden door buitensporige stress op de passieve elementen

•Stijvere spieren → grotere passieve krachtwerking → hoger risico op spierschade

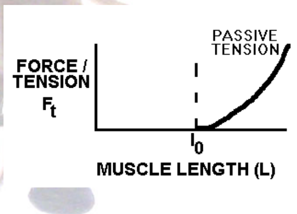

22

- Gevolgen van hypermobiele structuren
  - Excessieve flexibiliteit kan zowel een risicofactor zijn voor blessures als een nadeel voor prestatie
  - Hypermobiele structuren vereisen eerder stabilisatie dan extra mobilisatie-oefeningen

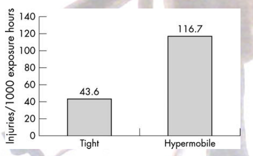

Uit: Stewart and Burden, 2004)

23

- Effecten van stretching
  - Elasticiteit van de pees stijgt onmiddellijk na stretching

Table 2 The measured parameters before and after stretching and isometric contractions (mean ± SD)

	MVC (Nm)	Stiffness (N mm^{-1})	Hysteresis (%)
Stretching			
Before	113 ± 7	24.0 ± 5.9	19.4 ± 9.2
After	111 ± 11	22.1 ± 5.3*	13.8 ± 8.7*
Isometric			
Before	115 ± 9	23.3 ± 5.5	19.8 ± 8.3
After	83 ± 12*	17.1 ± 5.2*	17.6 ± 9.4

*Significantly different from before.

Uit: Kubo et al., 2002

24

– Direct na stretching is er een shift in de angle-torque relatie: meer kracht kan geproduceerd worden bij grotere spierlengtes.

➢ Meer weerstand tegen excessieve spierverlenging

➢ Nog proefondervindelijk aan te tonen

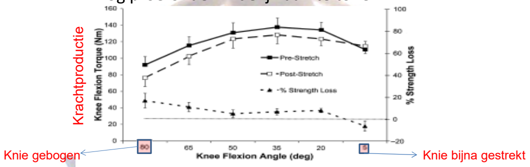

FIGURE 2—Isometric knee flexion torque prior to (*solid line, filled boxes*) and after (*dashed line, open boxes*) six passive hamstring stretches. Torque was measured at six different knee flexion angles from short muscle length (80°) to long muscle length (5°). Stretch × angle $P < 0.01$ (mean ± SE displayed). Percent strength loss at each angle is also displayed (*dashed line, closed triangles*) and graphed on the right *y*-axis (*dotted line* indicates zero strength loss); angle effect $P < 0.01$.

Uit: McHugh and Nesse, 2008

25

– Na langdurige stretching (3 weken – Kubo et al. 2002) verlaagt de viscositeit van de pees: efficiëntere werking tijdens activiteiten met inbegrip van stretch-shortening cycle (structuren worden eerst op rek gebracht en daarna verkorten ze)

Table 1. *Measured variables before and after stretching training*

	Trained		Control	
	Before	After	Before	After
MVC, Nm	131 ± 17	132 ± 20	130 ± 19	128 ± 18
Flexibility index, Nm/deg	1.43 ± 0.33	1.24 ± 0.30*	1.40 ± 0.29	1.42 ± 0.32
L_{max}, mm	25.1 ± 4.4	25.2 ± 3.8	25.0 ± 3.9	24.7 ± 4.3
Stiffness, N/mm	28.1 ± 4.9	27.4 ± 4.8	27.7 ± 4.5	28.2 ± 4.8
Hysteresis, %	19.9 ± 11.8	12.5 ± 9.5*	18.6 ± 10.2	19.1 ± 11.4

Values are means ± SD. MVC, maximal voluntary contraction; L_{max}, maximal tendon elongation. *Significantly lower than before, $P < 0.05$.

Uit: Kubo et al., 2002

26

– Hogere energie-absorberende werking van pezen
→ hoge stress op pezen zal minder gauw de maximale energie-absorberende capaciteit van pezen bereiken

→ minder transfer van energie naar het contractiele apparaat

➢ Lager risico op blessures in zowel spieren als pezen

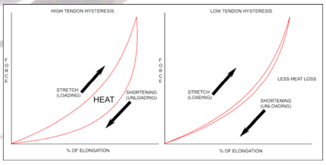

27

Richtlijnen

➢ Stretching vóór de prestatie: dynamisch mag, als deel van de aërobe opwarming, statisch niet (afkoeling, verminderde krachtproductie)

➢ Stretching leidt tot verminderde spierkracht

➢ DUS: geen statische stretching vóór prestatie, wel regelmatig stretchen

28

- Selecteer spieren die tijdens die bepaalde sport extra kwetsbaar zijn
- Stretching verschilt interindividueel: afhankelijk van mobiliteit van structuren
- Stretching na de prestatie: statisch is aangeraden.
  - Veiliger
  - Spieren zijn in verkorte toestand door inspanning. Belangrijk om spieren terug op oorspronkelijke lengte te brengen
- **Voorbeeld: iliopsoas – hamstrings - quadriceps**

29

Voorbeelden in de literatuur

Effectiviteit?	Auteur (jaar)	Studie design	Studiegroep
Stretching			
Effectief	Amako et al. (2003)	Non-RCT	Militairen
	Hartig and Henderson (1999)	Non-RCT	Militairen
	Verrall et al. (2005)	Non-RCT	Australian Footballers
Niet effectief	Pope et al. (2000)	RCT	Militairen

30

4. Krachttraining

- Voorste kruisband-blessures
  - Spieronevenwicht
    - Unilateraal (binnen hetzelfde been): hogere quadriceps activatie relatief tot hamstring activatie
    - Bilateraal: beendominantie (meer in dominante been)
- Spierblessures
  - Spieronevenwicht (unilateraal)
    - Onvermogen van de excentrische krachtwerking in de hamstrings om de concentrische krachtwerking in de quadriceps af te remmen

31

- Enkel verstuiking
  - Spieronevenwicht
    - Lagere dorsiflexie/plantairflexie ratio (tenen optrekken/tenen neerwaarts)

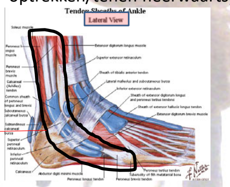

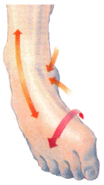

    - Beendominantie: meer in dominante been
  - Zwakke Peronei leiden tot onstabiele enkels

32

Mogelijke oplossingen

- Concentrisch - excentrische hamstringtraining
  - Vergroot de maximale kracht
  - Deel van de functionele spierwerking
  - Blessure vaak tijdens excentrische werking
  - **Voorbeelden**
- Plyometrie (spierverlenging gevolgd door contractie)
  - Musculoskeletale en neuromusculaire aanpassingen
  - Totnogtoe niet geïsoleerd getest
- Krachttraining enkelmusculatuur
  - Verhoogde proprioceptie **(Voorbeeld: m. tibialis – voorzijde onderbeen)**
- Sprongtraining: functioneel

33

Voorbeelden in de literatuur

Effectiviteit?	Auteur (jaar)	Studie design	Studiegroep
Krachttraining			
Effectief	Askling et al. (2003)	RCT	Voetballers
	Croisier et al. (2008)	Non-RCT	Voetballers
Niet effectief	Gabbe et al. (2006)	RCT	Australian Footballers

34

5. Balanstraining

- Lichaam uit balans → meer krachten rond knie- en enkelgewricht → risico op blessures
- Meer variatie in houdingsstabiliteit → verhoogde intersegmentele gewrichtskrachten → meer krachten ontwikkeld rond articulaire, ligamentaire en musculaire structuren

35

- Enkelverstuikingen
  - Correlatie tussen verhoogde postural sway (uitwijking lichaamsmassapunt in tweebenige stand) en verhoogde kans op enkelverstuikingen

TABLE 3. The occurrence of ankle joint injury among soccer players with pathological and normal values on stabilometric recording.

	Total	No ankle joint injury	Ankle joint injury	% ankle joint injuries
Pathologic stabilometric values	29	17	12	42
Normal stabilometric values	98	87	11	11

Uit: Tropp et al., 1984

- Knieblessures
  - Totnogtoe niet gecorreleerd met verhoogde postural sway of lage proprioceptie

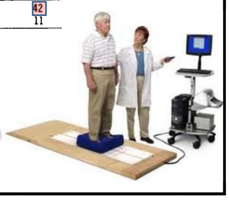

36

Vormen van balanstraining

- Geen materiaal
  (Voorbeeld opbouw)
- Balance Semi Globe
- Foam Pad
- Balance Board
- Sportspecifiek
  (Voorbeeld)

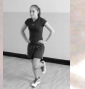
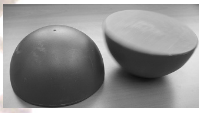
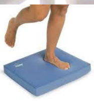
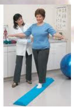
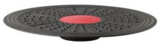
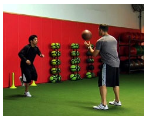

37

Voorbeelden in de literatuur

Effectiviteit?	Auteur (jaar)	Studie design	Studiegroep
Balanstraining			
Effectief	Caraffa et al. (1996)	Non-RCT	Voetballers
	Cumps et al. (2007)	Non-RCT	Basketballers
	Emery et al. (2005)	RCT	Humaniorastudenten
	McGuine and Keene (2006)	RCT	Basketballers en voetballers
	Verhagen et al. (2004)	RCT	Volleyballers
Niet effectief	Malliou et al. (2004)	RCT	Voetballers
	Söderman et al. (2000)	RCT	Voetbalsters

38

6. Core stability training

= stabiliteit in de regio van buik en onderrug

- Cruciaal als basis voor bewegingen van zowel bovenste als onderste ledematen (OL)
  - Musculus transversus abdominis (TA) is sleutelcomponent in core stability
  - TA is ook eerste gemobiliseerde spier bij bewegingen van de OL
    → Neurale systeem coördineert en preactiveert deze spier voor houdingsaanpassingen
- Lichaam uit balans → meer krachten rond knie- en enkelgewricht → risico op blessures

39

- ≠ spieren rond kniegewricht hebben oorsprong in regio van buik en lage rug
  DUS: verlaagde controle van rompmusculatuur
  → foute landingsmechanismen
  → verhoogde valguskrachten rond knie
- Core stability training → verhoogde heup abductiekracht en –recrutering
  → verhoogde controle van OL-alignement
  → verlaagde knie valgusbeweging en –belasting bij rompverplaatsingen tijdens sportactiviteiten

40

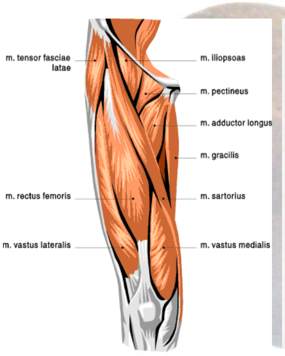

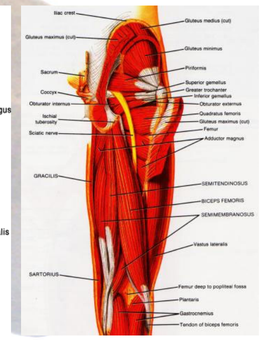

41

Richtlijnen

- Vooral rompuithouding (core endurance) belangrijk in preventie
  - Isometrische spieracties (spieren blijven op dezelfde lengte), lage belasting, lange periodes van spanning **(Voorbeeld)**
- Core stability
  - Aanpassingen van traditionele oefeningen: onstabiele oppervlakken, vrije gewichten, unilateraal, oefeningen met rotationele component met medecinebal
  - **(Voorbeeld)**

42

Voorbeelden in de literatuur

- Totnogtoe niet geïsoleerd getest
- MAAR:

  "Factors related to core stability predict risk of athletic knee, ligament and ACL injuries" (Zazulak et al., 2007)

43

7. Multiple interventie

- Meest effectieve en allesomvattende manier van blessurepreventie is een combinatie van twee of meerdere van voorgaande elementen
- Moeilijk te identificeren welk van de componenten effectief was in preventie van blessures
- Voorbeeld: basketbal

44

Voorbeelden in de literatuur

Effectiviteit?	Auteur (jaar)	Studie design	Studiegroep
Multiple interventie			
Effectief	Arnason et al. (2008)	Non-RCT	Voetballers
	Bahr et al. (1997)	Non-RCT	Volleyballers
	Bixler and Jones (1992)	Pseudo-RCT	American footballers
	Brooks et al. (2006)	Non-RCT	Rugby spelers
	Cahill et al. (1978)	Non-RCT	Voetballers
	Elphinston and Hardman (2006)	Non-RCT	Netbal spelers
	Emery et al. (2007)	RCT	Basketballers
	Emery and Meeuwisse (2010)	RCT	Voetballers
	Gilchrist et al. (2008)	RCT	Voetbalsters
	Heidt et al. (2000)	RCT	Voetbalsters
	Hewett et al. (1999)	Non-RCT	Volleybalsters, basketbalsters, voetbalsters
	Jakobsen et al. (1994)	Non-RCT	Lopers
	Junge et al. (2002)	Non-RCT	Voetballers
	[illegible]	[illegible]	[illegible]

45

Effectiviteit?	Auteur (jaar)	Studie design	Studiegroep
Multiple interventie			
Niet effectief	Brushoj et al. (2008)	RCT	Militairen
	Collard et al. (2010)	RCT	Schoolkinderen
	Engebretsen et al. (2008)	RCT	Voetballers
	Knapik et al. (2006)	Non-RCT	Militairen
	Myklebust et al. (2003)	Non-RCT	Handbalsters
	Petersen et al. (2005)	Non-RCT	Handbalsters
	Pfeiffer et al. (2006)	Non-RCT	Volleybalsters, basketbalsters, voetbalsters
	Steffen et al. (2008)	RCT	Voetbalsters
	Van Mechelen et al. (1993)	RCT	Lopers

46

8. Schoeisel aangepast aan de sportactiviteit

- "Footwear has been modified to suit the needs of individual disciplines. Here, too, the use of new materials has resulted in the production of specific shoes for every type of sport, athlete and surface and **for the prevention of accidents"**

  Benazzo et al. (1999)

47

9. Vermijden van hervalblessures

- 25-30% zijn hervalblessures
- Beïnvloedende factoren
  - Aard van de revalidatie
  - Opvolging van het revalidatieprogramma
  - Kwaliteit van herstel dat wordt bereikt

➢ Juist doorverwijzen!
  - ➢ Sportmedisch specialist op het HILO
  - ➢ Maandag- of dinsdagnamiddag
  - ➢ Terugbetalingstarief
  - ➢ Afspraak via Lennert Goossens

48

10. Luister naar je lichaam!

49

HOT TOPIC:
"Scheenbeenvliesontsteking"
Het mysterie ontrafeld??

50

Blessures aan het onderbeen

- Zowel in AJ 2010-2011 (22.9%) als in AJ 2011-2012 (23.7%) de meest voorkomende geblesseerde regio bij 1[e] bach studenten hilo
- Langdurige hinder/inactiviteit:
  - 2010-2011: 62.5% >1 week, 54.2% > 2 weken inactief
  - 2011-2012: 78.4% >1 week, 69.7% > 2 weken inactief
- Vaak herval:
  - 2010-2011: 24% hervalblessures
  - 2011-2012: 30.4% hervalblessures

51

Scheenbeenvliesontsteking

- Term wordt vaak ten onrechte in de mond genomen
- Zeer moeilijke diagnose, daarom nieuwe term "exercise related lower leg pain" (ERLLP)
- Omvat shin splint, shin pain, medial tibial stress syndrome (MTSS), periostitis, compartment syndrome en stress fracturen

52

Risicofactorenanalyse: moeizaam

- MTSS
  - Atleten met MTSS hebben lagere uithouding van plantairflexoren
  - Nog geen oorzaak-gevolg relatie bepaald
- Shin splint
  - Training van dorsiflexoren vermindert de pijn bij atleten met shin splint
  - Mogelijks ook preventief effect

53

- ERLLP
  - Overdreven pronatie en verlengde eversie met hogere belasting mediale zijde
  - Verhoogde re-inversie en verhoogde laterale afrol
  - **Verzwakte heup-abductorkracht!** (Verrelst et al. 2013)
  - Preventie
    - ➢ Pronatie uitslag en mediale drukverdeling minimaliseren
      MAAR: pronatie is integraal deel vd voetafrol
      DUS: individuele aanpassingen: orthoses, antipronatieschoenen
    - ➢ Gluteus medius (en maximus) optrainen **(Voorbeeld)**

➔ Totnogtoe geen evidenties voor effectiviteit van 1 of meerdere strategieën.

54

Waarom moet ik actief meewerken aan “NO GAIN WITH PAIN”?

- Minder blessures!
  - ➢Betere slaagkansen
  - ➢Langere sportcarrière/gezonde levensstijl
- Ontwikkeling tot betere/polyvalente/atletische sporters (Myer et al., 2005)
  - ➢Betere slaagkansen
  - ➢Betere prestaties in sport

55

Blessurepreventie → betere prestaties (Myer et al., 2005)

- 41 ♀ basketters, voetballers, volleyballers
- 6 weken multiple blessurepreventie
  - – Plyometrie, Core stability en balans, Weerstandstraining, Snelheidstraining
- ↗ 1RM squat
- ↗ 1RM bench press
- ↗ single leg hop-and-hold for distance
- ↗ double leg vertical jump
- ↘ sprinttijden

56
